# Determination of interatomic coupling between two-dimensional crystals using angle-resolved photoemission spectroscopy

**DOI:** 10.1038/s41467-020-17412-0

**Published:** 2020-07-17

**Authors:** J. J. P. Thompson, D. Pei, H. Peng, H. Wang, N. Channa, H. L. Peng, A. Barinov, N. B. M. Schröter, Y. Chen, M. Mucha-Kruczyński

**Affiliations:** 1 Department of Physics, University of Bath, Claverton Down, Bath BA2 7AY UK; 2 Chalmers University of Technology, Department of Physics, Gothenburg, SE-412 96 Sweden; 3 Clarendon Laboratory, Department of Physics, University of Oxford, Oxford, OX1 3PU UK; 4 Center for Nanochemistry, Beijing National Laboratory for Molecular Sciences, Peking University, Beijing, 100871 China; 5 Department of Physics, University of Warwick, Coventry, CV4 7AL UK; 6 Elettra-Sincrotrone Trieste ScPA, Trieste, 34149 Italy; 7 Swiss Light Source, Paul Scherrer Institute, Villigen, 5232 Switzerland; 8 Centre for Nanoscience and Nanotechnology, University of Bath, Claverton Down, Bath BA2 7AY UK

**Keywords:** Condensed-matter physics, Nanoscale materials, Electronic properties and devices, Electronic properties and devices, Physics

## Abstract

Lack of directional bonding between two-dimensional crystals like graphene or monolayer transition metal dichalcogenides provides unusual freedom in the selection of components for vertical van der Waals heterostructures. However, even for identical layers, their stacking, in particular the relative angle between their crystallographic directions, modifies properties of the structure. We demonstrate that the interatomic coupling between two two-dimensional crystals can be determined from angle-resolved photoemission spectra of a trilayer structure with one aligned and one twisted interface. Each of the interfaces provides complementary information and together they enable self-consistent determination of the coupling. We parametrise interatomic coupling for carbon atoms by studying twisted trilayer graphene and show that the result can be applied to structures with different twists and number of layers. Our approach demonstrates how to extract fundamental information about interlayer coupling in a stack of two-dimensional crystals and can be applied to many other van der Waals interfaces.

## Abstract (web summary)

Here, the authors show that the interatomic coupling between two layers of a 2D crystal can be determined by studying the angle-resolved photoemission spectra of a trilayer structure with one aligned and one twisted interface, and obtain the inter-atomic coupling for carbon atoms in twisted trilayer graphene.

## Introduction

Following the isolation of graphene (a layer of carbon atoms arranged in regular hexagons) in 2004^1^, many other atomically thin two-dimensional crystals have been produced and can be stacked in a desired order on top of each other. In contrast to conventional heterostructures, in which chemical bonding at interfaces between two materials modifies their properties and requires lattice matching for stability, stacks of two-dimensional crystals are held together by weak forces without directional bonding. As a result, any two of these materials can be placed on top of each other, providing extraordinary design flexibility^2–4^. Moreover, subtle changes in atomic stacking, especially the angle between the crystallographic axes of two adjacent layers, can have big impact on the properties of the whole heterostructure, with examples including the observation of Hofstadter’s butterfly^5,6^ and interfacial polarons^7^ in graphene/hexagonal boron nitride heterostructures, interlayer excitons in transition metal dichalcogenide bilayers^8,9^, appearance of superconductivity in magic-angle twisted bilayer graphene^10,11^ and explicit twist-dependence of transport measurements in rotatable heterostructures^12–14^. Phenomena like these arise because the misalignment of two crystals changes the atomic registry at the interface and hence tunes the spatial modulation of interlayer interaction. Consequently, understanding the coupling between two two-dimensional materials at a microscopic level is crucial for efficient design of van der Waals heterostructures.

**Fig. 1:**
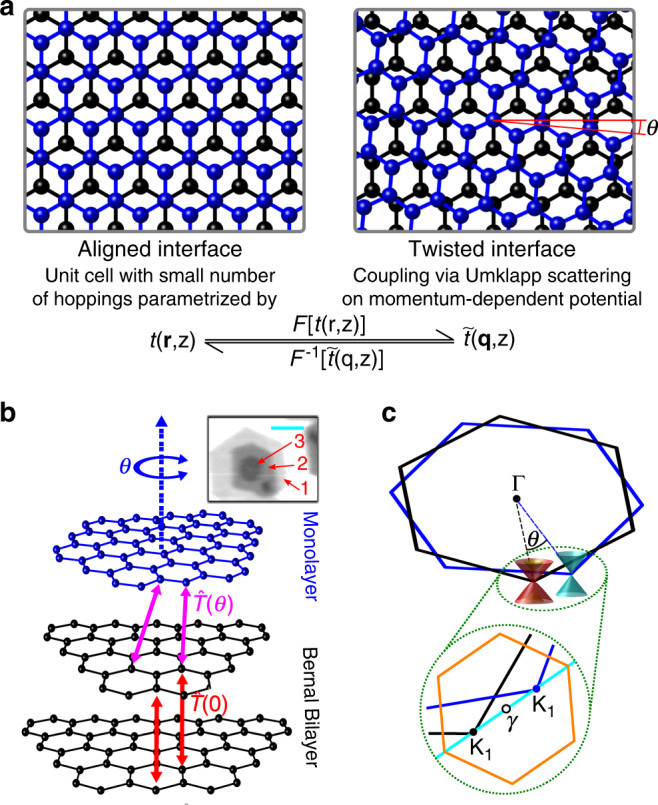
Aligned vs twisted interfaces in van der Waals heterostructures. **a** Comparison of aligned and twisted interfaces for two-dimensional crystals and the descriptions in the real and reciprocal spaces used in this article. Blue and black balls indicate atoms in the top and bottom layer, respectively. **b** Schematic of twisted trilayer graphene with monolayer (blue) stacked at an angle on top of a Bernal bilayer (black). The red and purple arrows indicate the interlayer couplings for the Bernal and twisted interfaces which are captured by the blocks $\hat{T}(0)$ and $\hat{T}(\theta )$, respectively, in the Hamiltonian in Eq. (1). Inset shows photoemission intensity from copper substrate which is attenuated by graphene layers above, providing a measure of graphene layer number. The red arrows indicate each of the graphene layers in the trilayer stack and the cyan line corresponds to the distance of 10 μm. **c** Brillouin zones of the Bernal bilayer (black) and rotated monolayer (blue) with bilayer and monolayer graphene low-energy electronic spectra shown in the vicinities of one set of the Brillouin zone corners. The inset depicts in orange the superlattice Brillouin zone and the cyan line indicates the *k*-space path cuts along which are presented in Figs. 2a and 3.

The impacts of a twisted interface and modulated interlayer coupling on the electronic properties of two-dimensional crystals include band hybridisation^15–17^, band replicas and minigaps due to scattering on moiré potential^15,18,19^, charge transfer and vertical shifting of bands^17,20,21^ as well as changes of the effective masses^17,20^. Variations in the interlayer coupling as a function of the twist angle, *θ*, were probed for example using photoluminescence, Raman and angle-resolved photoemission (ARPES) spectroscopies^20,22–24^. Here, we use the last of those methods to image directly the electronic bands in trilayer graphene with one perfect and one twisted interface. From our data, we extract the interatomic coupling, *t*(**r**, *z*), describing coupling between two carbon atoms separated by a vector **r**_3D_ = (**r**, *z*) = (*x*, *y*, *z*). Such coupling functions, usually based on comparisons to ab initio calculations, can be used to determine electron hoppings in tight-binding^25,26^ and continuum^27,28^ models of corresponding van der Waals interfaces at any twist angle. We show that *t*(**r**, *z*) determined purely by measurements on one of the structures accurately describes electronic dispersions obtained for stacks with different *θ* and number of layers, providing an experimentally verified set of parameters to model twistronic graphene. Our approach makes use of the fact that a trilayer structure is the thinnest stack that can contain both a perfect and twisted interface. The former, due to translational symmetry, can be straightforwardly described in the real space using *t*(**r**, *z*). At the same time, the impact of the moiré pattern formed at the latter can be captured in the reciprocal space by considering scattering by moiré reciprocal vectors on the momentum-dependent potential $\tilde{t}({\bf{q}},z)$ which is a two-dimensional Fourier transform ${\mathcal{F}}[t({\bf{r}},z)]$ of *t*(**r**, *z*) (see the comparison of the two cases in Fig. 1a). As a consequence, this method should enable determination of interatomic couplings for all van der Waals interfaces for which moiré effects were observed.

## Results

### ARPES of twisted trilayer graphene

We grew our graphene trilayers on copper foil using chemical vapour deposition^29,30^. The inset of Fig. 1b shows the intensity map of copper *d*-band photoelectrons which are attenuated differently by the overlying graphene layers depending on their number. This provides means to identify all of the layers in our stack, shown in the inset with different shades of grey and indicated with the red arrows. As depicted schematically in the main panel of Fig. 1b, the bottom two layers form a Bernal bilayer (2L) while the crystallographic axes of the top monolayer (1L) are rotated by an angle *θ* with respect to those of the layer underneath. As a result, the Brillouin zones corresponding to the bilayer and monolayer are also rotated with respect to each other, Fig. 1c. We focus here on the vicinity of one set of the corners of the two Brillouin zones, which we denote **K**_2_ and **K**_1_, for the bilayer and monolayer, respectively. The separation between these two points, dependent on the twist angle, defines an effective superlattice Brillouin zone, indicated in orange in the inset of Fig. 1c.

**Fig. 2:**
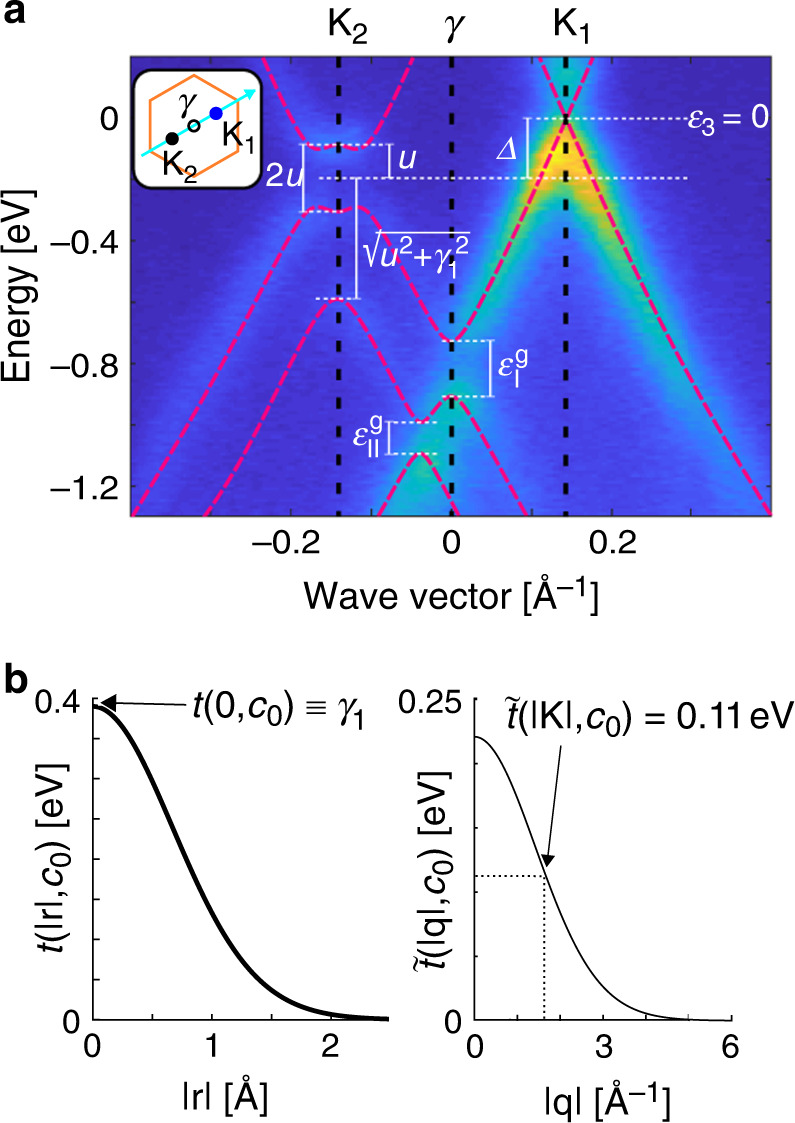
Angle-resolved photoemission spectra and interatomic coupling. **a** ARPES intensity for twisted trilayer with twist *θ* = 9. 6^∘^, measured along the direction connecting Brillouin zone corners **K**_2_ and **K**_1_ as shown in Fig. 1c and indicated in the inset. The calculated miniband structure along the same path is shown with red dashed lines. White dashed and solid lines indicate the important energies used to fit the parameters of our theoretical model. **b** Left: Real-space interatomic coupling, *t*(∣**r**∣, *c*_0_), as a function of distance ∣**r**∣ between carbon atoms, as given in Eq. (3) with parameter values from Table 1. Right: Two-dimensional Fourier transform $\tilde{t}(| {\bf{q}}| ,{c}_{0})$ of the interatomic coupling, *t*(∣**r**∣, *c*_0_), as a function of wave vector ∣**q**∣.

In Fig. 2a, we present ARPES intensity along a cut in the *k*-space connecting **K**_2_ and **K**_1_, with the energy reference point set to the linear crossing (Dirac point) at **K**_1_. Close to each corner, the intensity reflects the low-energy band structures of unperturbed 2L and 1L. Because the bilayer flake is below the monolayer, signal from the former is attenuated due to the electron escape depth effect. In between the two spectra, coupling of the two crystals leads to anticrossings of the bands and opening of minigaps (marked as ${\varepsilon }_{{\rm{I}}}^{{\rm{g}}}$ and ${\varepsilon }_{{\rm{II}}}^{{\rm{g}}}$ in the figure). As the size of the superlattice Brillouin zone depends on the twist angle, the energy positions of the minigaps also depend on *θ*. Moreover, the magnitudes of the minigaps depend on the interlayer coupling between the bilayer and monolayer and also, in principle, vary with *θ*. However, fundamentally, all of the features in our spectrum originate in interactions between carbon atoms, be it in the same or different layers, at the twisted or aligned interface. This provides us with an opportunity to study the interatomic coupling *t*(**r**, *z*) in carbon materials.

### Parametrising carbon-carbon interaction potential

In order to understand our data, we use a generic Hamiltonian for a van der Waals heterostructure comprised of three layers of the same two-dimensional crystal

1 $${\hat{H}}=\left(\begin{array}{ccc}{\hat{H}}_{0}\left(0,\Delta -u\right)&\hat{T}(0)&0\\ {\hat{T}}^{\dagger }(0)&{\hat{H}}_{0}\left(0,\Delta +u\right)&\hat{T}(\theta )\\ 0&{\hat{T}}^{\dagger }(\theta )&{\hat{H}}_{0}\left(\theta ,0\right)\end{array}\right).$$

In this Hamiltonian, the diagonal block, ${\hat{H}}_{0}\left({\theta }_{i},{\varepsilon }_{i}\right)$ describes the *i*-th layer at a twist angle *θ*_*i*_, with on-site energies of atomic sites in this layer, *ε*_*i*_. Here, because only the relative twist between any two adjacent layers is important, we have *θ*_1_ = *θ*_2_ = 0 and *θ*_3_ = *θ*. Also, our choice of energy reference point is equivalent to *ε*_3_ = 0 and we introduce potential energy difference, 2*u* = *ε*_1_ − *ε*_2_, as well as average energy, Δ = (*ε*_1_ + *ε*_2_)/2, of layers 1 and 2 (the charge transfer between the copper foil and the graphene layers giving rise to *u* ≠ Δ ≠ 0 is discussed in more detail in ref. ^29^). For graphene, the intralayer blocks ${\hat{H}}_{0}$ can be straight-forwardly described using a tight-binding model^31^ for a triangular lattice with two inequivalent atomic sites, *A* and *B*, per unit cell and nearest neighbour coupling between them *γ*_0_ ≡ −*t*(**r**_*AB*_, 0), where **r**_*AB*_ is a vector connecting neighbouring *A* and *B* atoms with the carbon-carbon bond length ∣**r**_*AB*_∣ = 1.46 Å.

Of more importance for us, however, are the off-diagonal blocks $\hat{T}({\theta }_{i}-{\theta }_{i-1})$ which capture the twist-dependent interlayer interactions between adjacent layers (we neglect the interaction between the bottom and the top layers which is at least an order of magnitude weaker^32^). As the bottom two layers are stacked according to the Bernal stacking, a real-space description of the interlayer interaction block $\hat{T}(0)$ is possible with the leading coupling *t*(0, *c*_0_) ≡ *γ*_1_, with interlayer distance *c*_0_ = 3.35 Å, due to atoms with neighbours directly above or below them, as shown in Fig. 1a^33^. In contrast, we describe the coupling between the twisted layers, *i* = 2, 3, in the reciprocal space based on electron tunnelling from a state with wave vector **k** in layer 2 to a state with wave vector ${\bf{k}}^{\prime}$ in layer 3 with the requirement that crystal momentum is conserved^34,35^, ${\bf{k}}+{\bf{G}}={\bf{k}}^{\prime} +{\bf{G}}^{\prime}$, where **G** and ${\bf{G}}^{\prime}$ are the reciprocal vectors of layers 2 and 3, respectively. The strength of a given tunnelling process is set by the two-dimensional Fourier transform, ${\mathcal{F}}[t({\bf{r}},z)]=\tilde{t}({\bf{q}},z)$, of the real-space coupling *t*(**r**, *z*) so that

2 $${\hat{T}}(\theta )=	\, \mathop{\sum }\limits_{{\bf{G}},{{\bf{G}}}^{\prime}}\tilde{t}({\bf{k}}+{\bf{G}},z)\\ 	\, \times \left(\begin{array}{cc}{{\rm{e}}}^{{\rm{i}}{\bf{G}}\cdot {\boldsymbol{\tau }}}&{{\rm{e}}}^{{\rm{i}}({\bf{G}}{\hat{R}}_{\theta }+{{\bf{G}}}^{\prime})\cdot {\boldsymbol{\tau }}}\\ 1&{{\rm{e}}}^{{\rm{i}}{\hat{R}}_{\theta }{{\bf{G}}}^{\prime}\cdot {\boldsymbol{\tau }}}\end{array}\right){\delta }_{{\bf{k}}+{\bf{G}},{{\bf{k}}}^{\prime}+{{\bf{G}}}^{\prime}},$$

where ***τ*** = (−∣**r**_*AB*_∣, 0) and ${\hat{R}}_{\theta }$ is a matrix of clockwise rotation by angle *θ* (see Supplementary Note 1 for more details on the construction of the Hamiltonian $\hat{H}$).

The uniqueness of a trilayer with one perfect and one twisted interface (as exemplified in Fig. 1a for the case of graphene) lies in the fact that the Hamiltonian $\hat{H}$ contains interlayer blocks based on both the real-space ($\hat{T}(0)$) and reciprocal-space ($\hat{T}(\theta )$) descriptions which provide complementary information and at the same time are related to each other because of the Fourier transform connection between *t*(**r**, *z*) and $\tilde{t}({\bf{q}},z)$. Because of this, comparison of the photoemission data with the spectrum calculated based on Eq. (1) provides more information about the interatomic coupling *t*(**r**, *z*) than structures with one type of interface only. For our graphene trilayer, we compute the miniband spectrum of $\hat{H}$ (see Methods for more details) assuming a Slater-Koster-like two-centre ansatz for *t*(**r**, *z*)^25^,

3 $$t({\bf{r}},z)=	\, t(| {\bf{r}}| ,z)\\ =	\, {V}_{\pi }({\bf{r}},z)\left(1-\frac{{z}^{2}}{| {{\bf{r}}}_{{\rm{3}}{\rm{D}}}{| }^{2}}\right)+{V}_{\sigma }({\bf{r}},z){\left(\frac{z}{| {{\bf{r}}}_{{\rm{3}}{\rm{D}}}| }\right)}^{2},\\ {V}_{\pi }({\bf{r}},z)=	\, {-}{\gamma }_{0}\exp \left[-{\alpha }_{\pi }(| {{\bf{r}}}_{{\rm{3}}{\rm{D}}}| -| {{\bf{r}}}_{AB}| )\right],\\ {V}_{\sigma }({\bf{r}},z)=	\, {\gamma }_{1}\exp \left[-{\alpha }_{\sigma }(| {{\bf{r}}}_{{\rm{3}}{\rm{D}}}| -{c}_{0})\right],$$

where *V*_*π*_ and *V*_*σ*_ represent the strength of the *π* and *σ* bonding^36^, respectively, and *α*_*π*_ and *α*_*σ*_ their decay with increasing interatomic distance.

In fitting our numerical results to the experimental data in Fig. 2a, we first determine the position of 1L Dirac point what sets the *ε* = 0 reference point. We then use the electronic band gap at **K**_2_ to fix the electrostatic potential 2*u* and position the bilayer neutrality point halfway in the gap, establishing the potential energy shift Δ. We obtain the in-plane nearest neighbour hopping *γ*_0_ from the slope of the 1L linear dispersion close to the Dirac point at **K**_1_ while the direct interlayer coupling *γ*_1_ is set by the splitting of the 2L lower valence band from the neutrality point at **K**_2_. Finally, the decay constants *α*_*π*_ and *α*_*σ*_ are found numerically using the constraints that (i) the magnitudes of the gaps ${\varepsilon }_{{\rm{I}}}^{{\rm{g}}}$ and ${\varepsilon }_{{\rm{II}}}^{{\rm{g}}}$ in Fig. 2a match the experimental data and (ii) in the limit of *θ* = 0, $\hat{T}(\theta )$ from Eq. (2) converges to the real-space form of $\hat{T}(0)$ as used for coupling between the Bernal stacked layers (see Supplementary Note 2 for further discussion).

**Fig. 3:**
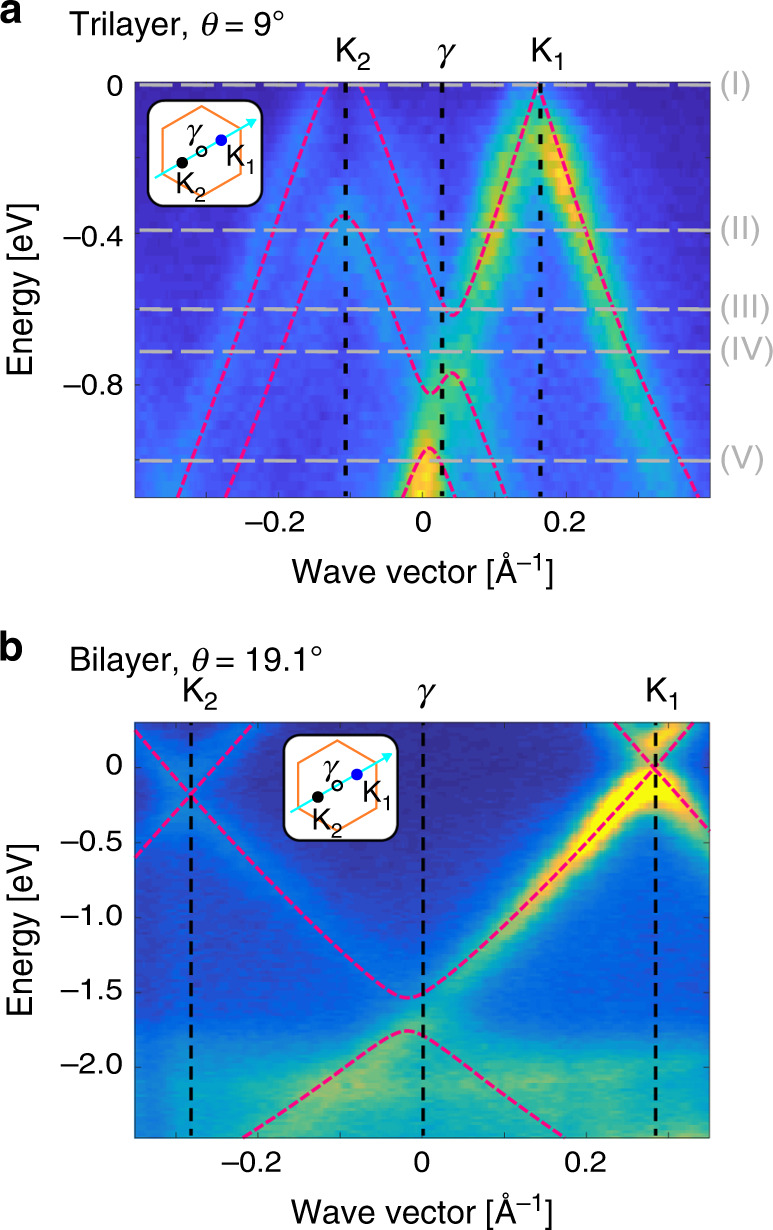
Modelling stacks with different twists and layer numbers. Comparison of the ARPES intensity and the calculated electronic band structure (obtained using the parameter set in Table 1 and shown with red dashed lines) for **a** twisted trilayer with *θ* = 9^∘^ and **b** twisted bilayer with *θ* = 19.1^∘^, both measured along the direction connecting Brillouin zone corners **K**_2_ and **K**_1_ as shown in Fig. 1c and indicated in the inset. In **a**, the grey dashed lines, labelled (I)-(V), indicate energies for which constant-energy ARPES intensity maps are presented in Fig. 4.

The miniband spectrum resulting from our model is shown in red dashed lines in Fig. 2a, the functions *t*(∣**r**∣, *c*_0_) and $\tilde{t}(| {\bf{q}}| ,{c}_{0})$ are plotted in Fig. 2b and the corresponding values of the parameters *γ*_0_, *γ*_1_, *α*_*π*_ and *α*_*σ*_ are summarised in Table 1. The interatomic potential we obtain decays more rapidly in the real space (and hence slower in the reciprocal space) than suggested by computational results^25^. Importantly, parametrization of *t*(**r**, *z*) does not depend on the twist angle and so should be applicable to other graphene stacks with twisted interfaces. It also does not depend on the doping level because, for the relevant range of electric fields, the electrostatic energies Δ and *u* do not modify the electron hoppings. At the same time, once these energies are determined for a particular stack, their influence on the band structure (shifting of the positions and magnitudes of anticrossings) is captured through the Hamiltonian $\hat{H}$. To confirm applicability of a single parametrization of *t*(**r**, *z*) to different graphene stacks, we compare in Fig. 3 the miniband spectra computed using the parameters from Table 1 to ARPES intensities measured along a similar **K**_2_-**K**_1_*k*-space cut for, in Fig. 3a, a trilayer with *θ* = 9^∘^ and, in Fig. 3b, twisted bilayer with *θ* = 19.1^∘^. Our model describes the bands of both of the structures well, despite changes in the twist angle, number of layers, potentials *u* and Δ (which vary with growth conditions and thickness of the stack^29^ and are determined for each structure individually) and the magnitudes of minigaps.

**Table 1:** Parametrization of *t*(∣**r**∣, *z*) Values of fitting parameters describing the carbon-carbon potential *t*(∣**r**∣, *z*) from Eq. (3).

Fitting constants			
*γ*_0_ [eV]	*γ*_1_ [eV]	*α*_*π*_ [Å^−1^]	*α*_*σ*_ [Å^−1^]
2.95	0.39	3.39	6.78

### Probing electron wave function

**Fig. 4:**
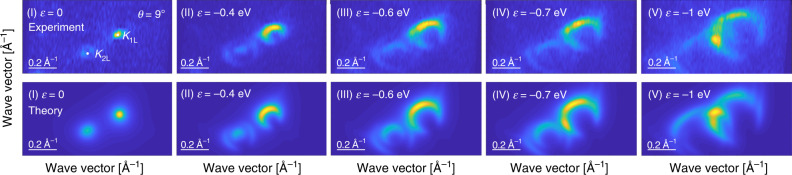
Wave function symmetry in ARPES. Comparison of experimental (top row) and theoretical (bottom row) constant-energy ARPES intensity maps for twisted trilayer graphene with *θ* = 9^∘^ for energies indicated with grey dashed lines in Fig. 3. The intensities are normalised in each row.

We assess the accuracy of our parametrization of the interatomic potential, *t*(**r**, *z*), further by modelling directly the ARPES intensity data (we use approach developed in ref. ^37^ and applied to the graphene/hexagonal boron nitride heterostructure in ref. ^38^; see Methods and Supplementary Note 3 for further details). In graphene materials, interference of electrons emitted from different atomic sites within the unit cell provides additional information about the electronic wave function^37^. This is best visualised by ARPES intensity patterns at constant electron energy, which we present, both as obtained experimentally (top row) and simulated theoretically (bottom row), in Fig. 4 for the trilayer sample with *θ* = 9^∘^ and energies indicated with grey dashed lines in Fig. 3. For the map at the energy *ε* = 0, the two spots of high intensity indicate the positions of the valleys **K**_1_ and **K**_2_. For energies 0 < *ε* < −0.6 eV, the bilayer and monolayer dispersions are effectively uncoupled. The crescent-like intensity pattern in the vicinity of **K**_1_ reflects the pseudospin of *n* = 1 (evidence of Berry phase of *π*^39^) of electrons in monolayer graphene. In contrast, in bilayer graphene, the low-energy band hosts massive chiral fermions^40^ with pseudospin *n* = 2 so that the outer ring pattern in the vicinity of **K**_2_ displays two intensity maxima, feature best visible in panel (II). Because in our model all electron hoppings are generated naturally by *t*(**r**, *z*), agreement of our ARPES simulation with experimental data provides confirmation that our model and parametrization of the interatomic coupling *t*(**r**, *z*) leads to the correct band structure. Finally, panels (III)-(V) in Fig. 4 show the constant-energy maps in the vicinity of the minigaps which open due to hybridisation of the bilayer and monolayer bands. The merging of 1L and 2L contours in panel (III) leads to a van Hove singularity and an associated peak in the electronic density of states, similarly to the case of twisted bilayer graphene^15^ and discussed also for twisted trilayer graphene^29^ (in the latter, the position of the van Hove singularity is established by tracking the minigap; the former is caused by saddle points in the electronic dispersion as the bands flatten at the anticrossings and so every minigap is accompanied by a van Hove singularity). Overall, our simulated patterns correctly reflect the evolution of the minigap as a function of energy and wave vector as well as the measured photocurrent intensity.

## Discussion

Our parametrization of *t*(**r**, *z*) is applicable to a wide range of twist angles, including the magic-angle regime^10,34^ as well as the 30^∘^-twisted bilayer graphene quasicrystal^41,42^. To mention, it yields the *k*-space interlayer coupling at the graphene Brillouin zone corner **K**, $\tilde{t}(| {\bf{K}}| ,{c}_{0})=0.11$ eV. This agrees with the values used in effective models of the low-twist limit of twisted bilayer graphene^27,34,35,43^ which require $\tilde{t}(| {\bf{K}}| ,{c}_{0})$ as the only parameter. Overall, our form of *t*(**r**, *z*) decays more rapidly in the real space (and hence slower in the reciprocal space) than usually assumed. This might explain the discrepancy between theory and experimental ARPES intensities of Dirac cone replicas observed for the case of 30^∘^-twisted bilayer graphene in ref. ^41^.

As we have shown, the same interatomic coupling *t*(**r**, *z*) can be used in graphene structures with different number of layers as, similarly to the case of perfect graphite and other layered materials, coupling to the nearest layer dominates the interlayer couplings. The continuum approach has been applied extensively to model the graphene/graphene interface, including to predict the existence of the magic angle^34^. Hence, in Supplementary Figure 1, we use our results to simulate ARPES spectra for twist angles in the vicinity of the magic angle, *θ* ≈ 1.1^∘^, and show qualitative agreement with the recent experimental data^44,45^. The continuum model was also used successfully to interpret experimental observations in graphene on hexagonal boron nitride^5^ as well as homo- and heterobilayers of transition metal dichalcogenides^46,47^. Our approach allows for experimental parametrization of the interatomic coupling *t*(**r**, *z*) for each of these interfaces as well as for others for which influence of neighbouring crystals can be approximated by considering the harmonics of the moiré potential^43,48–52^. To comment, previous studies suggest that adapting our model to stacks of transition metal dichalcogenides requires taking into account changes in the interlayer distance as a function of the twist angle^20^. Moreover, in contrast to graphene, for which the part of $\tilde{t}({\bf{q}},z)$ most relevant to modelling twisted interfaces is that for **q** pointing to the Brillouin zone corner, **q** ≈ **K**, for transition metal dichalcogenides more significant changes due to interlayer coupling occur in the vicinity of the Γ point. In multilayers of 2H semiconducting dichalcogenides MX_2_ (M = Mo, W, and X = S, Se), coupling of the degenerate states at the Γ point built of transition metal ${d}_{{z}^{2}}$ and chalcogen *p*_*z*_ orbitals leads to their hybridisation and splitting which drives the direct-to-indirect band gap transition^53,54^. Using the form of *t*(**r**, *z*) suggested in ref. ^26^ for chalcogen *p*_*z*_-to-*p*_*z*_ hopping (which dominates the interlayer coupling) in transition metal disulfides and diselenides, we computed the corresponding $\tilde{t}({\bf{q}},z)$ and obtained an estimate of $\tilde{t}(\Gamma ,{c}_{{\rm{X}}-{\rm{X}}}) \sim 1.2$ eV for interlayer nearest neighbour distance between chalcogen sites, *c*_X−X_ ≈ 3 Å. Taking into account the fractional contribution of the *p*_*z*_ orbitals to the top valence band states at Γ in a monolayer^26^, we obtain coupling between two such states in bilayer  ~0.4 eV. This, in turn, suggests band splitting of  ~0.8 eV, in qualitative agreement with observations^53–55^. This supports the idea that our model can accurately describe and parametrise interatomic coupling between materials other than graphene.

Experimentally, our approach requires fabrication of trilayer (or thicker) stacks with one twisted and one perfect interface in order to benefit from the complementarity of the information obtained from self-consistent real- and momentum-space description of the interfaces. However, to note, building on the observations of superconductivity in magic-angle twisted bilayer graphene^10,11^, structures containing both a twisted and a perfect interface like twisted trilayer graphene^56,57^, double bilayer graphene^58–63^ or double bilayer WSe_2_^64^ recently attracted attention on its own due to observation of correlated electronic behaviour. Our approach provides one of the avenues to build an experimentally validated single-particle base to study such effects. It could be, in principle, also applied to stacks of different materials, as long as one of the interfaces is commensurate and can be described in the real space in a tight-binding-like fashion. Finally, apart from continuum models, the interatomic coupling *t*(**r**, *z*) can also be used directly in large scale tight-binding calculations for commensurate twist angles^25,26,65–67^.

## Methods

### ARPES measurements

The ARPES measurements were performed at the Spectromicroscopy beamline at the Elettra synchrotron (Trieste, Italy). Before measurements, the samples were annealed at 350^∘^ for 30 minutes. The experiment was then performed at a base pressure of 10^−10^ mbar in ultrahigh vacuum and at the temperature of 110 K. We used photons with energy of 74 eV and estimate our energy and angular resolution as 50 meV and 0.5^∘^, respectively. For each sample, we determined the twist angle *θ* by measuring the distance between the Brillouin zone corners **K**_2_ and **K**_1_ which depends on the twist angle, $| {{\bf{K}}}_{2}-{{\bf{K}}}_{1}| =\frac{8\pi }{3\sqrt{3}| {{\bf{r}}}_{AB}| }\sin \frac{\theta }{2}$. Further comments on experimental analysis of ARPES intensity are provided in Supplementary Note 4.

### Theoretical calculations

We write the Hamiltonian $\hat{H}$ in Eq. (1) in the basis of sublattice Bloch states constructed of carbon *p*_*z*_ orbitals *ϕ*(**r**_3D_)^31^,

$${\left|{\bf{k}},X\right\rangle }_{l}=\frac{1}{\sqrt{N}}\mathop{\sum }\limits_{{{\bf{R}}}_{l}}{{\rm{e}}}^{{\rm{i}}{\bf{k}}\cdot ({{\bf{R}}}_{l}+{{\boldsymbol{\tau }}}_{X,l})}\phi ({{\bf{r}}}_{{\rm{3D}}}-{{\bf{R}}}_{l}-{{\boldsymbol{\tau }}}_{X,l}),$$

where **k** is electron wave vector, *X* = *A*, *B* is the sublattice, **R**_*l*_ are the lattice vectors of layer *l* and ***τ***_*X*,*l*_ points to the site *X* in layer *l* within the unit cell selected by **R**_*l*_. We include in the basis all states coupled to **k** through $\hat{T}(\theta )$ which are less than a distance $\frac{28\pi }{3\sqrt{3}{r}_{AB}}\sin \frac{\theta }{2}$ away from it, compute the matrix elements of $\hat{H}$ in this truncated basis and diagonalize the resulting matrix numerically. In order to simulate the ARPES intensity, we project the eigenstates of the moiré Hamiltonian, $\hat{H}$, on a plane-wave-like final state (see Supplementary Note 3 for more details and ref. ^38^ for a detailed discussion of this approach for the case of graphene on hexagonal boron nitride). We determine the broadening of the ARPES signal as well as the decay constant for the intensity of Bernal bilayer signal by fitting to the experimental data.

## Supplementary information

Supplementary Information

## Data Availability

The data used in this study are available from the University of Bath data archive at 10.15125/BATH-00864^68^.
