# Supplementary material for: Determination of interatomic coupling between two-dimensional crystals using angle-resolved photoemission spectroscopy
Source: Nat Commun. 2020 Jul 17;11:3582. doi: 10.1038/s41467-020-17412-0 (PMC7367817; doi:10.1038/s41467-020-17412-0)
Supplement: Supplementary file 1 — Supplementary Information [file 41467_2020_17412_MOESM1_ESM.pdf]

Supplementary Information: Determination of interatomic coupling in twisted graphene using angle-resolved photoemission spectroscopy

Thompson et al.

Supplementary Note 1: Electronic minibands of twisted trilayer graphene

As mentioned in the main text, we aim to construct our Hamiltonian $\hat{H}$ based on blocks corresponding to intra- and interlayer couplings,

$$\hat{H} = \begin{pmatrix} \hat{H}_0\left(0, \Delta - u\right) & \hat{T}(0) & 0 \\ \hat{T}^{\dagger}(0) & \hat{H}_0\left(0, \Delta + u\right) & \hat{T}(\theta) \\ 0 & \hat{T}^{\dagger}(\theta) & \hat{H}_0\left(\theta, 0\right) \end{pmatrix}. \quad (1)$$

Above, the diagonal blocks $\hat{H}_0\left(\theta_i, \varepsilon_i\right)$ describe consecutive graphene layers (starting from the top left for layer 1) at a twist angle θ_i and with on-site energies of atomic sites in this layer, ε_i. Without loss of generality, we choose $\theta_1 = 0$ (we measure twists with respect to the crystallographic directions of layer 1) and, from our structure, $\theta_2 = 0$ immediately follows. In contrast, we set the reference point for energy as the on-site energies of layer 3 equal to zero, $\varepsilon_3 = 0$. From the definitions of the energies Δ and u in the main text, we get

$$\varepsilon_1 = \Delta - u, \quad \varepsilon_2 = \Delta + u.$$

Consider now the sublattice Bloch states constructed of carbon p_z orbitals $\phi(\mathbf{r}_{3\mathrm{D}}) \equiv \phi(\mathbf{r}, z)$,

$$\left|\mathbf{k}, X\right\rangle_l = \frac{1}{\sqrt{N}} \sum_{\mathbf{R}_l} e^{i\mathbf{k}\cdot(\mathbf{R}_l + \boldsymbol{\tau}_{X,l})} \phi(\mathbf{r} - \mathbf{R}_l - \boldsymbol{\tau}_{X,l}, z - z_l), \quad (2)$$

where $\mathbf{k} = (k_x, k_y)$ is electron wave vector, $X = A, B$ is the sublattice, $\mathbf{R}_l$ are the lattice vectors of layer l, $\boldsymbol{\tau}_{X,l}$ points to the site X in layer l within the unit cell selected by $\mathbf{R}_l$ and z_l defines the position of layer l along the z-axis. In the basis $\{\left|\mathbf{k}, A\right\rangle_l, \left|\mathbf{k}, B\right\rangle_l\}$, the intralayer blocks take a familiar tight-binding form [1],

$$\hat{H}_0(\theta_l, u_l) = \begin{pmatrix} u_l & -\gamma_0 f(\hat{R}_{\theta_l}\mathbf{k}) \\ -\gamma_0 f^*(\hat{R}_{\theta_l}\mathbf{k}) & u_l \end{pmatrix}, \quad (3)$$

$$f(\mathbf{k}) = \exp\left(\frac{ik_y a}{\sqrt{3}}\right) + 2\exp\left(-\frac{ik_y a}{2\sqrt{3}}\right)\cos\left(\frac{k_x a}{2}\right),$$

where $a = \sqrt{3}|\mathbf{r}_{AB}|$ is the lattice constant of graphene. Similarly, the interlayer coupling block between layers 1 and 2 which are perfectly aligned and stacked according to the Bernal stacking [2],

$$\hat{T}(0) = \begin{pmatrix} 0 & 0 \\ \gamma_1 & 0 \end{pmatrix}. \quad (4)$$

Let us now consider the interface between layers 2 and 3. The positions of the j-th and m-th sublattices in these layers can be expressed as

$$\begin{aligned} \mathbf{R}_j &= n_1\mathbf{a}_1 + n_2\mathbf{a}_2 + \boldsymbol{\tau}_{j,2}, \\ \mathbf{R}^{'}_m &= n^{'}_1\hat{R}_\theta\mathbf{a}_1 + n^{'}_2\hat{R}_\theta\mathbf{a}_2 + \hat{R}_\theta\boldsymbol{\tau}_{m,3}, \end{aligned} \tag{5}$$

where $\mathbf{a}_1$ and $\mathbf{a}_2$ are the primitive lattice vectors of layer 2 and $n_1, n_2, n^{'}_1, n^{'}_2 \in \mathbb{Z}$. For no twist, the two layers would be Bernal-stacked and that prescribes the relation between $\boldsymbol{\tau}_{j,2}$ and $\boldsymbol{\tau}_{m,3}$. Here, we choose

$$\begin{aligned} \boldsymbol{\tau}_{A,2} &= -(0, a/\sqrt{3}), \;\; \boldsymbol{\tau}_{B,2} = (0,0), \\ \boldsymbol{\tau}_{A,3} &= (0,0), \qquad\quad\;\; \boldsymbol{\tau}_{B,3} = (0, a/\sqrt{3}). \end{aligned} \tag{6}$$

Following earlier work [3, 4], the interlayer coupling matrix element ${}_3\langle \mathbf{k}', m, |\hat{T}|\mathbf{k}, j\rangle_2$ between sublattice Bloch states on layers 2 and 3 can be written as

$${}_3\langle \mathbf{k}', m, |\hat{T}|\mathbf{k}, j\rangle_2 = \sum_{\mathbf{G},\mathbf{G}'} \tilde{t}(\mathbf{k}+\mathbf{G}, c_0) e^{-i\mathbf{G}\cdot\boldsymbol{\tau}_{j,2}} e^{i\mathbf{G}'\cdot\hat{R}_\theta\boldsymbol{\tau}_{m,3}} \, \delta_{\mathbf{k}+\mathbf{G},\mathbf{k}'+\mathbf{G}'}. \tag{7}$$

The matrix element above can be understood in the following way: (1) the Kronecker delta term, $\delta_{\mathbf{k}+\mathbf{G},\mathbf{k}'+\mathbf{G}'}$, expresses conservation of crystal momentum and determines the set of momenta on the top and bottom layers which are coupled (these are effectively momenta $\mathbf{k}$ and $\mathbf{k}'$ which are offset by a moiré reciprocal lattice vector $\mathbf{g} = \mathbf{G}' - \mathbf{G}$); (2) the phase $e^{i(\mathbf{G}'\cdot\hat{R}_\theta\boldsymbol{\tau}_{m,3} - \mathbf{G}\cdot\boldsymbol{\tau}_{l,2})}$ describes the phase factor associated with the coupling of orbitals m and l as a result of translations by reciprocal lattice vectors in each layer. Qualitatively, these phases describe a continuous transition between regions of AA, AB and BA-type stacking present in the moiré pattern between twisted graphene layers. Finally, the strength of the coupling, $\tilde{t}(\mathbf{k}+\mathbf{G}, c_0)$, is prescribed by the (total) momentum of the electron tunnelling between the layers 2 and 3.

By writing all four matrix elements in the form of a 2×2 matrix, we obtain Eq. (2) of the main text,

$$\hat{T}(\theta) = \sum_{\mathbf{G},\mathbf{G}'} \tilde{t}(\mathbf{k}+\mathbf{G}, c_0) \times \begin{pmatrix} e^{i\mathbf{G}\cdot\boldsymbol{\tau}} & e^{i(\mathbf{G}+\hat{R}_\theta\mathbf{G}')\cdot\boldsymbol{\tau}} \\ 1 & e^{i\hat{R}_\theta\mathbf{G}'\cdot\boldsymbol{\tau}} \end{pmatrix} \delta_{\mathbf{k}+\mathbf{G},\mathbf{k}'+\mathbf{G}'}, \tag{8}$$

where we only use $\boldsymbol{\tau} \equiv \boldsymbol{\tau}_{A,2} = -(0, a/\sqrt{3})$.

In order to obtain the energy dispersion for a given $\mathbf{k}$, we include into our Hamiltonian all states coupled to $\mathbf{k}$ through $\hat{T}(\theta)$ which are less than a distance $\frac{28\pi}{3\sqrt{3}r_{AB}} \sin\frac{\theta}{2}$ away from

it, compute the matrix elements of $\hat{H}$ in this truncated basis and diagonalize the resulting matrix numerically.

Supplementary Note 2: Self-consistency check for interlayer coupling blocks

We use comparison between the limit $\theta \to 0$ of the interlayer block $\hat{T}(\theta)$ written in the reciprocal space, Supplementary Equation (8), and the $\hat{T}(0)$ block corresponding to interlayer coupling for a perfect Bernal bilayer, Supplemetary Equation (4), as a check of self-consistency of our model. In this limit, the superlattice Brillouin zone shrinks to a single point and the reciprocal vectors of both layers become identical.

Now, consider an electron state with wave vector $\mathbf{k}$ in the vicinity of the valley corner $\mathbf{K}$, $\mathbf{k} \approx \mathbf{K}$, as well as the states with wave vectors $\mathbf{k} + \mathbf{G}$. These can be grouped according to their distance from the origin [for example, the first group is given by $\mathbf{K}$, $\mathbf{K} - \frac{2\pi}{a}(1, \frac{1}{\sqrt{3}})$ and $\mathbf{K} - \frac{2\pi}{a}(1, -\frac{1}{\sqrt{3}})$]. Hence, the terms in the sum in Supplementary Equation (8) can be grouped according to the same coupling magnitudes, $\tilde{t}(\mathbf{k} + \mathbf{G}, c_0) \equiv \tilde{t}(|\mathbf{k} + \mathbf{G}|, c_0) \approx \tilde{t}(|\mathbf{K} + \mathbf{G}|, c_0)$. Because of the rapid decay of $\tilde{t}(\mathbf{k}, c_0)$ as a function of $|\mathbf{k}|$, the sum converges. Taking note of the partial cancellation of the phase factors, in the limit $\theta \to 0$ equivalence of the descriptions in the real and reciprocal spaces requires that

$$\hat{T}(\theta \to 0) \approx 3\left[\tilde{t}(\mathbf{K}, c_0) + \tilde{t}(2\mathbf{K}, c_0) + 2\tilde{t}(\sqrt{7}\mathbf{K}, c_0) + ...\right] \begin{pmatrix} 0 & 0 \\ 1 & 0 \end{pmatrix} = \begin{pmatrix} 0 & 0 \\ \gamma_1 & 0 \end{pmatrix}, \qquad (9)$$

to reproduce the interlayer coupling in Bernal bilayers. As $\gamma_1 \equiv t(0, c_0)$, this condition provides us with additional constraints on the behaviour of $t(\mathbf{r}, z)$ and its Fourier transform $t(\mathbf{k}, z)$.

Supplementary Note 3: Theoretical model of ARPES intensity

Using Fermi's golden rule, we write ARPES intensity as [5, 6]

$$I \propto \sum_i |M_{f,i}|^2 \, \delta(\omega + \varepsilon_{i,\mathbf{k}} - W - \varepsilon_{\mathbf{p}_e}), \tag{10}$$

where $M_{f,i}$ is the matrix element describing transition of the electron from the initial state in the crystal in band i to the final state f, ω is the energy of the incident photon, $\varepsilon_{i,\mathbf{k}}$ is the energy of an electron in the crystal in band i and with wave vector $\mathbf{k}$, $\varepsilon_{\mathbf{p}_e}$ is the energy of the photoelectron with momentum $\mathbf{p}_e$ and W is the work function of graphene. Within the dipole approximation,

$$M_{f,i} \propto \langle \text{final} | \, \hat{A} \cdot \hat{p} \, | \mathbf{k}, i \rangle \, , \tag{11}$$

where $\hat{A}$ is the vector potential of the incident photon, $\hat{p}$ is the momentum operator, $|\text{final}\rangle$ stands for the final state of the photoelectron and $|\mathbf{k}, i\rangle$ denotes the wave function of the electron in the crystal. The latter is a linear combination of the sublattice Bloch states, Supplementary Equation (2), corresponding to wave vectors connected by a superlattice reciprocal vector $\mathbf{g} = \mathbf{G}' - \mathbf{G}$,

$$|\mathbf{k}, i\rangle = \sum_{\mathbf{g}} \sum_{l,X} c^{\mathbf{g},i}_{X,l}(\mathbf{k}) \, |\hat{R}_{\theta_l}(\mathbf{k}+\mathbf{g}), X\rangle_l \, , \tag{12}$$

with the coefficients $c^{\mathbf{g},i}_{X,l}(\mathbf{k})$ provided by diagonalization of the Hamiltonian $\hat{H}$, Supplementary Equation (1). Here, $\mathbf{g}$ is the moiré reciprocal superlattice vector. We approximate the final state with a plane wave (justified for incident photon energies above 50 eV [7]) with momentum $\mathbf{p}_e = (\mathbf{p}_e^{\parallel}, p_e^{\perp})$, so that

$$M_{f,i} \propto \sum_{\mathbf{g}} \sum_{l,X} c^{\mathbf{g},i}_{X,l}(\mathbf{k}) \, \langle e^{\frac{i}{\hbar}\mathbf{p}_e^{\parallel}\cdot\mathbf{r}} e^{\frac{i}{\hbar}p_e^{\perp}z} | \, \hat{A} \cdot \hat{p} \, | \hat{R}_{\theta_l}(\mathbf{k}+\mathbf{g}), X\rangle_l \, .$$

Following from the chiral properties of the graphene wave function, the light-matter interaction $\hat{A} \cdot \hat{p}$ leads to an angle-dependent phase difference between the distinct atomic orbitals in the matrix element, $e^{i\varphi_{X,l}}$ [8–10] (note that this includes the effect of changing the sign of γ_1 [10]). Hence,

$$\begin{aligned} M_{f,i} &\propto \sum_{\mathbf{g}} \sum_{l,X} e^{i\varphi_{X,l}} c^{\mathbf{g},i}_{X,l}(\mathbf{k}) \, \langle e^{\frac{i}{\hbar}\mathbf{p}_e^{\parallel}\cdot\mathbf{r}} e^{\frac{i}{\hbar}p_e^{\perp}z} | \hat{R}_{\theta_l}(\mathbf{k}+\mathbf{g}), X\rangle_l \\ &= \sum_{\mathbf{g}} \sum_{l,X,\mathbf{G}_l} e^{i\varphi_{X,l}} c^{\mathbf{g},i}_{X,l}(\hat{R}_{-\theta_l}(\mathbf{p}_e^{\parallel}/\hbar + \mathbf{G}_l) - \mathbf{g}) e^{i\mathbf{G}_l\cdot\boldsymbol{\tau}_{X,l}} e^{-\frac{i}{\hbar}p_e^{\perp}z_l} \tilde{\phi}\left(\hat{R}_{\theta_l}(\mathbf{k}+\mathbf{g}) - \mathbf{G}_l, p_e^{\perp}/\hbar\right) , \end{aligned}$$

where

$$\tilde{\phi}\left(\mathbf{p}_e^{\parallel}/\hbar, p_e^{\perp}/\hbar\right) = \int d\mathbf{r}\, dz\, e^{-\frac{i}{\hbar}\mathbf{p}_e^{\parallel}\cdot\mathbf{r}} e^{-\frac{i}{\hbar}p_e^{\perp}z}\phi(\mathbf{r}, z),$$

is the Fourier transform of the p_z orbital $\phi(\mathbf{r}, z)$.

Due to the rotational symmetry of the p_z orbital, $\tilde{\phi}\left(\mathbf{p}_e^{\parallel}/\hbar, p_e^{\perp}/\hbar\right) = \tilde{\phi}\left(|\mathbf{p}_e^{\parallel}/\hbar|, p_e^{\perp}/\hbar\right)$. Moreover, for the given photon energy, ω, and work function, W, we have $p_e^{\perp} \gg |\mathbf{p}_e^{\parallel}|$ so that $\tilde{\phi}\left(\hat{R}_{\theta_l}(\mathbf{k}+\mathbf{g}) - \mathbf{G}_l, p_e^{\perp}/\hbar\right)$ can be approximated by a constant and dropped. Finally, in this work we only study points for which $\mathbf{G}_l = 0$. As a result,

$$I \propto \sum_i \left| \sum_{\mathbf{g}} \sum_{l,X} c_{X,l}^{\mathbf{g},i}(\hat{R}_{-\theta_l}\mathbf{p}_e^{\parallel} - \mathbf{g}) e^{i\varphi_{X,l}} e^{-\frac{i}{\hbar}p_e^{\perp} l c_0} \right|^2 \delta(\omega + \varepsilon_{i,\mathbf{k}} - W - \varepsilon_{\mathbf{p}_e}), \tag{13}$$

where we used the fact that $z_l = lc_0$. We combine both phases $\exp(i\varphi_{X,l})$ and $\exp(-\frac{i}{\hbar}p_e^{\perp}lc_0)$ into a single factor,

$$e^{i\varphi_{X,l}} e^{-\frac{i}{\hbar}p_e^{\perp} l c_0} = e^{i\alpha_{X,l}}, \tag{14}$$

which we fit to experiment. To note, the experimental data in this work suggests that the phase difference between the aligned graphene layers is approximately $e^{i\pi}$.

Finally, we model the Dirac delta in Supplementary Equation (13) with a Lorentzian

$$\delta(\omega + \varepsilon_{i,\mathbf{k}} - W - \varepsilon_{\mathbf{p}_e}) \to \frac{1}{\pi} \frac{\gamma}{(\omega + \varepsilon_{i,\mathbf{k}} - W - \varepsilon_{\mathbf{p}_e})^2 - \gamma^2}, \tag{15}$$

with half-width-half-maximum γ (the value of which, $\gamma \approx 0.17$ eV, we obtain by comparison to the experimental data).

Supplementary Note 4: Fitting experimental ARPES data

In most cases, our graphene samples are electron-doped so that the Dirac and neutrality points are located below the Fermi level. To determine the energy position of the Dirac and neutrality points, we (i) select a point in the reciprocal space close to the anticipated location of the Brillouin zone corners, (ii) find the average between binding energies of intensity maxima corresponding to states in the valence and conduction bands. To check consistency, we repeat this procedure for several reciprocal space points. Similarly, we look at ARPES intensity along a cut in the reciprocal space passing through the assumed position of the Dirac/neutrality point and exploit circular symmetry of the low-energy electronic dispersion to triangulate their position. Once the locations of the Brillouin zone corners for the Bernal bilayer and twisted monolayer are established, the twist angle can be obtained by measuring the distance in reciprocal space between them (as explained in the main text, this distance is directly governed by the twist angle).

As part of our data analysis, we extract the differences between on-site potentials on different graphene layers. For Bernal bilayer, we investigate the intensity at the corner of its Brillouin zone as a function of energy as the gap in the electronic dispersion is equal to $2|u|$ [2]. The neutrality point is located halfway in the gap. In turn, the energy shift between the neutrality point and the position of the Dirac point of layer 3 defines Δ.

Finally, in our theoretical analysis, we assume such an orientation of the graphene layers that one of the Brillouin zone corners of the first layer is located at $\mathbf{K} = \frac{4\pi}{3a}(1,0)$. In comparison, depending on the experimental geometry, the Brillouin zones of the graphene layers can be rotated arbitrarily about the Γ point. Once the positions of the Dirac/neutrality points have been determined, we apply a global rotation about the Γ point to map the positions of the neutrality points in the theoretical model onto the experimental features.

Supplementary Figure 1: Simulating ARPES spectra of small-angle twisted bilayer graphene

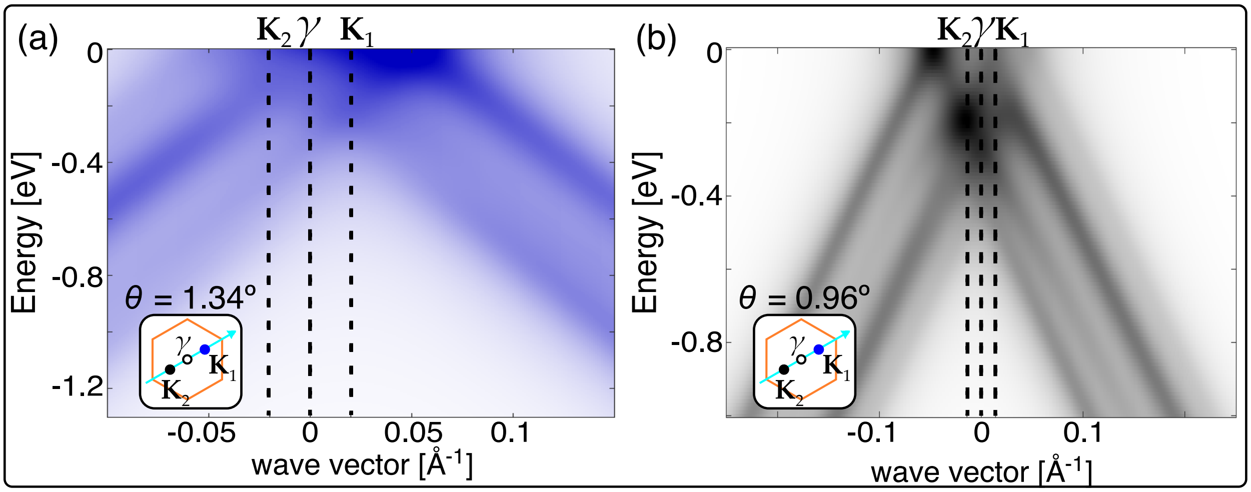

FIG. 1. **ARPES of twisted bilayer graphene close to magic angle.** Simulation of ARPES intensity for twisted bilayer graphene with (a) $\theta = 1.34°$ and (b) $\theta = 0.96°$ along the $\mathbf{K}_2$-$\mathbf{K}_1$ cut as introduced in the main text and to be compared with experimental data shown in Ref. [11] and [12], respectively. Above, we used our model with the parametrization of $t(\mathbf{r}, z)$ as presented in the main text. We choose the spectral broadening as well as wave vector and energy ranges to match the experimental data (unfortunately, neither of the experimental works zoom in on the flat band features). The results are in qualitative agreement with the presented experimental photoemission intensity. We do not make comparisons to the constant energy maps presented in [11] as it is unclear to us exactly what the symmetrization procedure is that is used to generate these maps.

Supplementary References

[1] Castro Neto, A. H., Guinea, F., Peres, N. M. R., Novoselov, K. S., and Geim, A. K. The electronic properties of graphene. *Reviews of Modern Physics* **81**, 109 (2009).

[2] McCann, E. and Falko, V. I. Landau-Level Degeneracy and Quantum Hall Effect in a Graphite Bilayer. *Physical Review Letters* **96**, 086805 (2006).

[3] Bistritzer, R., and MacDonald, A. H. Moiré bands in twisted double-layer graphene. *Proceedings of the National Academy of Sciences* **108**, 12233 (2011).

[4] Koshino, M. Interlayer interaction in general incommensurate atomic layers. *New Journal of Physics* **17**, 015014 (2015).

[5] Mucha-Kruczynski, M. et al. Characterization of graphene through anisotropy of constant-energy maps in angle-resolved photoemission. *Physical Review B* **77**, 195403 (2008).

[6] Mucha-Kruczynski, M., Wallbank, J. R., and Falko, V. I. Moiré miniband features in the angle-resolved photoemission spectra of graphene/hBN heterostructures. *Physical Review B* **93**, 085409 (2008).

[7] Gierz, I., Lindroos, M., Hochst, H., Ast, C. R., and Kern, K. Graphene Sublattice Symmetry and Isospin Determined by Circular Dichroism in Angle-Resolved Photoemission Spectroscopy. *Nano Letters* **12**, 3900 (2012).

[8] Liu, Y., Bian, G., Miller, T., and Chiang, T.-C. Visualizing Electronic Chirality and Berry Phases in Graphene Systems Using Photoemission with Circularly Polarized Light. *Physical Review Letters* **107**, 166803 (2011).

[9] Gierz, I., Henk, J., Hochst, H., Ast, C. R., and Kern, K. Illuminating the dark corridor in graphene: Polarization dependence of angle-resolved photoemission spectroscopy on graphene. *Physical Review B* **83**, 121408 (2011).

[10] Hwang, C. et al. Direct measurement of quantum phases in graphene via photoemission spectroscopy. *Physical Review B* **84**, 125422 (2011).

[11] Lisi, S. et al. Direct evidence for flat bands in twisted bilayer graphene from nano-ARPES. arXiv:2002.02289 (2020).

[12] Utama, M. I. B. et al. Visualization of the flat electronic band in twisted bilayer graphene near the magic angle twist. arXiv:1912.00587 (2019).
